# BRAF inhibitors stimulate inflammasome activation and interleukin 1 beta production in dendritic cells

**DOI:** 10.18632/oncotarget.25511

**Published:** 2018-06-19

**Authors:** Eva Hajek, Franziska Krebs, Rebekka Bent, Katharina Haas, Antje Bast, Ivo Steinmetz, Andrea Tuettenberg, Stephan Grabbe, Matthias Bros

**Affiliations:** ^1^ Department of Dermatology, University Medical Center of the Johannes Gutenberg University, Mainz, Germany; ^2^ Friedrich Loeffler Institute of Medical Microbiology, University Medicine Greifswald, Greifswald, Germany

**Keywords:** BRAF^V600E^ inhibitor, MEK inhibitor, dendritic cell, IL-1beta, inflammasome, Immunology

## Abstract

Melanoma is the most dangerous form of skin cancer with a growing incidence over the last decades. Fourty percent of all melanomas harbor a mutation in the signaling adaptor BRAF (V600E) that results in ERK hyperactivity as an oncogenic driver. In these cases, treatment with the BRAF^V600E^ inhibitors Vemurafenib (VEM) or Dabrafenib (DAB) coapplied with the MEK1/2 inhibitors Cobimetinib (COB) or Trametinib (TRA) can result in long-term suppression of tumor growth. Besides direct suppression of ERK activity, these inhibitors have been reported to also modulate tumor immune responses, and exert pro-inflammatory side effects such as fever and rash in some patients. Here we asked for potential effects of BRAF^V600E^ inhibitors on dendritic cells (DC) which are essential for the induction of adaptive anti-tumor responses. Both splenic and bone marrow-derived (BM) mouse dendritic cells (DC) up-regulated costimulator expression (CD80, CD86) in response to DAB but not VEM treatment. Moreover, DAB and to lesser extent VEM enhanced IL-1β (interleukin 1 beta) release by splenic DC, and by LPS-stimulated BMDC. We demonstrate that DAB and VEM activated the NLRC4/Caspase-1 inflammasome. At high concentration, DAB also induced inflammasome activation independent of Caspase-1. TRA and COB elevated MHCII expression on BMDC, and modulated the LPS-induced cytokine pattern. Immunomodulatory activity of DAB and VEM was also observed in human monocyte-derived DC, and DAB induced IL-1β in human primary DC. Altogether, our study shows that BRAF^V600E^ inhibitors upregulate IL-1β release by mouse and human DC which may affect the DC-mediated course of anti-tumor immune responses.

## INTRODUCTION

Until a few years ago, no effective drug was available for treatment of metastatic melanoma. This situation changed with the introduction of immunotherapeutics and targeted therapies for melanoma treatment, including antibody-based immunotherapeutic approaches that block the inhibitory CTLA4-CD80/CD86 and PD1-PD-L1 signaling axes apparent in all types of tumor progression (reviewed in [1].

In addition, mutagenome analysis identified a common point mutation within the BRAF (v-Raf murine sarcoma viral oncogene homolog B) protein (BRAF^V600E^) apparent in >40% of all melanoma cases [2]. BRAF serves as an adaptor kinase in the ERK (extracellular signal-regulated kinases) pathway that transmits stimulatory signals of receptor binding growth factors [3]. Activation of ERK results in cell cyle progression, and thereby cell proliferation [4]. In case of BRAF^V600E^, autoinhibiton of BRAF is prevented which results in hyperactivation of ERK. Hence, BRAF^V600E^ constitutes an oncogenic driver.

To terminate BRAF^V600E^-mediated constitutive ERK signaling in tumor cells, several small molecule inhibitors have been developed. Of these, Vemurafenib (VEM) and Dabrafenib (DAB) have been clinically approved for treatment of unresectable stage III and of stage IV melanoma [5]. However, tumor resistance towards BRAF^V600E^ inhibitor therapy was observed in about half of all patients after 6–8 months of treatment. By now, a number of resistance mechanisms that result in reactivation of ERK have been identified, including the induction of BRAF isoforms [6], amplification of the BRAF gene locus [7], and activating mutations of other signaling molecules within the ERK pathway [8]. To prevent reactivation of ERK signaling, small molecule inhibitors that target signaling adaptors downstream of BRAF have been introduced [9]. Indeed, treatment of patients with VEM plus the MEK inhibitor cobimetinib (COB) [10], and of DAB coapplied with the MEK inhibitor trametinib (TRA) [11] significantly prolonged progression-free and overall survival of melanoma patients as compared with the according BRAF^V600E^ inhibitor monotherapy.

These kinds of combination therapies were reported not only to inhibit the ERK pathway in tumor cells, but to also exert immuno-relevant effects that may significantly contribute to their clinical efficacy [12]. In this regard, treatment of melanoma cells *in vitro* with BRAF^V600E^ inhibitors resulted in elevated presentation of melanoma-associated antigens, irrespective of the melanoma BRAF mutation state [13]. In addition, treatment of melanoma with VEM was reported to inhibit BRAF^V600E^-induced IL-1 (interleukin 1) production by melanoma cells and thereby to diminish IL-1 dependent production of protolerogenic prostaglandins and PD-L1/2 in the tumor micro-environment [14]. Likewise, cotreatment of melanoma patients with VEM and COB was shown to reduce VEGFR expression at the tumor site [15]. Furthermore, effector T cells were reported to be stimulated via so-called paradoxical activation of ERK signaling by BRAF^V600E^ inhibitors [16]. In agreement, tumor specimens of patients under treatment with BRAF^V600E^ plus MEK inhibitors showed increased infiltration with cytotoxic T cells [15]. Altogether, these findings indicate that BRAF^V600E^ inhibitors may exert anti-tumor activity due to direct tumor-associated effects, but also via off target effects on immune cells.

Dendritic cells (DC) are crucial for the induction of adaptive anti-tumor T cell responses [17]. Here we asked for potential off target effects of VEM and DAB, and of the according MEK inhibitors COB and TRA on the immuno-phenotype of DC when applied at clinically relevant doses. We show that DAB alone partially activated splenic and bone marrow-derived (BM) mouse dendritic cells (DCs) as reflected by elevated costimulator expression (CD80, CD86) and strongly increased IL-1β levels. DAB enhanced both IL-1β mRNA expression and inflammasome activation via NLRC4. At high dose, DAB acted in a Caspase-1 independent manner via Caspase-8 to elevate IL-1β. TRA alone stimulated MHCII expression, and had no detrimental effect on DAB-dependent IL-1β induction. In contrast to DAB, VEM alone yielded no DC activation, but enhanced IL-1β as well, although at much lower level than observed for DAB. VEM enhanced IL-1β mRNA expression as well, and strictly required NLRC4/Caspase-1 inflammasome activation to induce IL-1β expression in BMDC. Both BRAF^V600E^ inhibitors also elevated IL-1β secretion by splenic DC.

## RESULTS

### DAB enhances expression of costimulators by DC, in synergy with TRA, and TRA alone elevates MHCII expression

Our study aimed to elucidate potential off-target effects of clinically relevant BRAF^V600E^ inhibitors on immune cells. Due to the dual role of DC for the maintenance of peripheral tolerance and the induction of primary immune responses we used mouse bone marrow-derived DC (BMDC) for subsequent experiments.

In melanoma therapy, BRAF^V600E^ inhibitors are commonly coapplied with an assigned MEK1/2 inhibitor to efficiently block ERK signaling in tumor cells and thus to provide development of resistance mechanisms. In this regard, DAB is administered in combination with Trametinib (TRA). Viability analysis showed that when applied alone both DAB and TRA (Supplementary Figure 1A) yielded no major cytotoxic effects on CD11c^+^ BMDC at concentrations below 10 µM at unstimulated state (upper panel) and when coapplied with LPS (lower panel). Coapplication of DAB in combination with TRA resulted in a more pronounced decrease in viability. In subsequent experiments non-toxic concentrations were used as indicated. Similar effects were obtained with VEM and COB, although these substances were somewhat more toxic when applied at higher doses (Supplementary Figure 1B).

**Figure 1:**
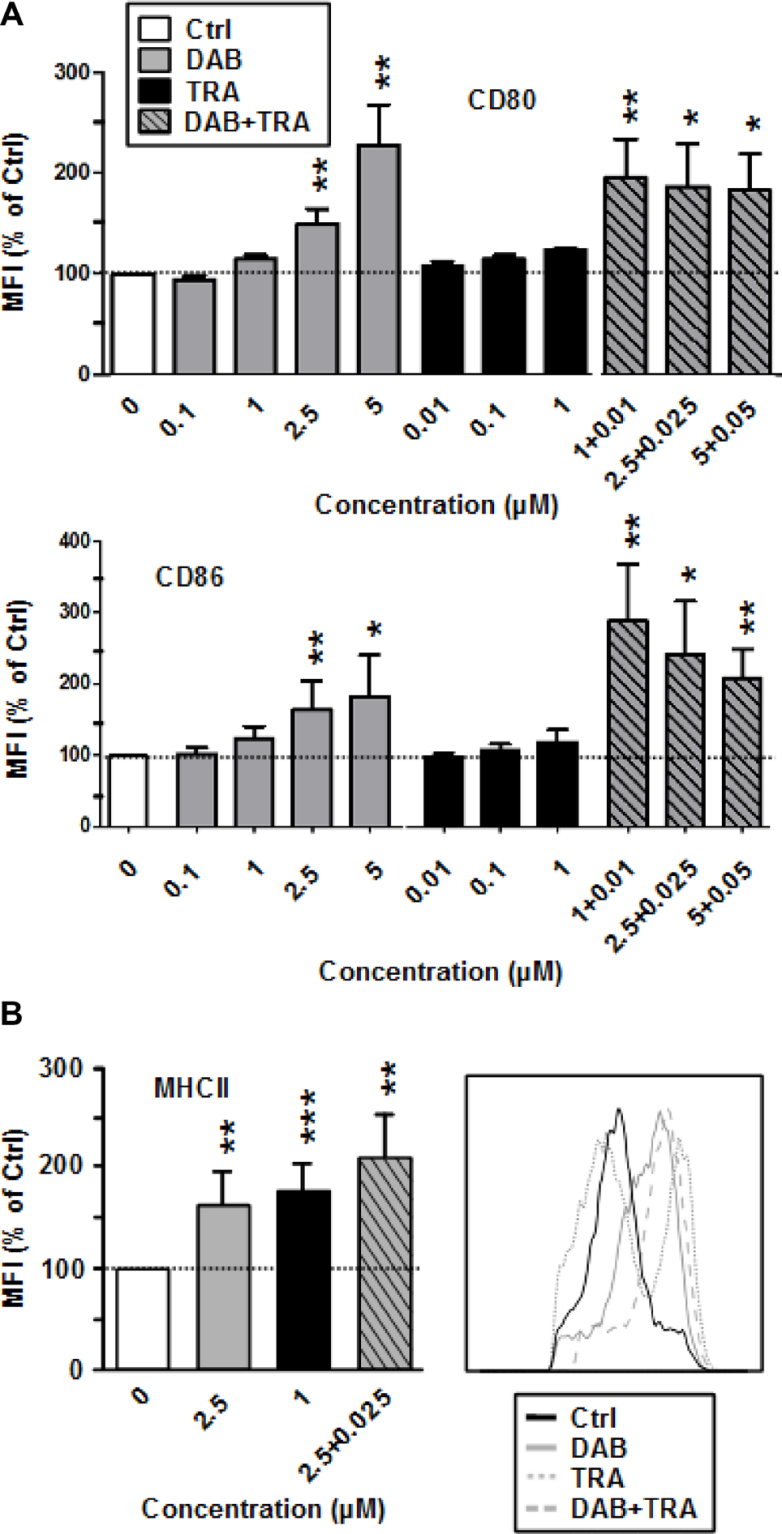
DAB elevates costimulator expression by BMDC, and TRA enhances MHCII expression DAB and Trametinib (TRA) were applied alone or in combination at the concentrations indicated to unstimulated BMDC. DMSO (1‰) served as a solvent control (Ctrl). After 24 h, expression of (**A**) CD80, CD86, and (**B**) of MHCII by CD11c^+^ BMDC was assessed by flow cytometry. (A, B) Data show the MFI of the respective surface marker on pre-gated CD11c^+^ BMDC, normalized to the expression in Ctrl, arbitrarily set to 100% in each experiment. Data represent the mean ± SEM of 3–4 independent experiments each. (B, *right panel*), Histogram of MHCII expression by CD11c^+^ BMDC after differential treatment is representative of 3 independent experiments each. (A, B) Statistical differences: ^*^versus control (Ctrl). ^*^p < 0.05, ^**^p < 0.01, ^***^p < 0.001.

DAB enhanced expression of CD86 as well as of CD80 (Figure 1A) and of MHCII (Figure 1B) at high (CD86: 5 µM) and intermediate (CD80, MHCII: 2.5 µM) doses. TRA had no effect on the expression of costimulators, but enhanced MHCII expression. More strikingly, DAB (1 µM) and TRA (0.01 µM) when coapplied at low concentrations upregulated expression of CD80 and CD86 at maximal extent. Coapplication of DAB and TRA stimulated MHC-II expression at similar extent as observed for application of either agent alone. DAB applied at non-toxic concentrations (Supplementary Figure 2A) also activated splenic antigen presenting cell (APC) populations as reflected by elevated CD86 expression (Supplementary Figure 2B). Stimulation of BMDC with LPS as an internal control resulted in upregulation of all surface activation markers monitored which was not strongly affected by additional application of DAB and TRA (not shown).

Altogether, these results indicate that DAB partially activates BMDC, enhanced by concurrent MEK1/2 inhibition.

### DAB strongly upregulates IL-1β production by unstimulated and LPS-stimulated DC, but diminishes other proinflammatory cytokines

DAB had no overall stimulatory effect on cytokine production by unstimulated BMDC, but dose-dependently induced IL-1β, which was further enhanced in response to cotreatment with DAB and TRA at highest concentration (Supplementary Figure 3).

**Figure 2:**
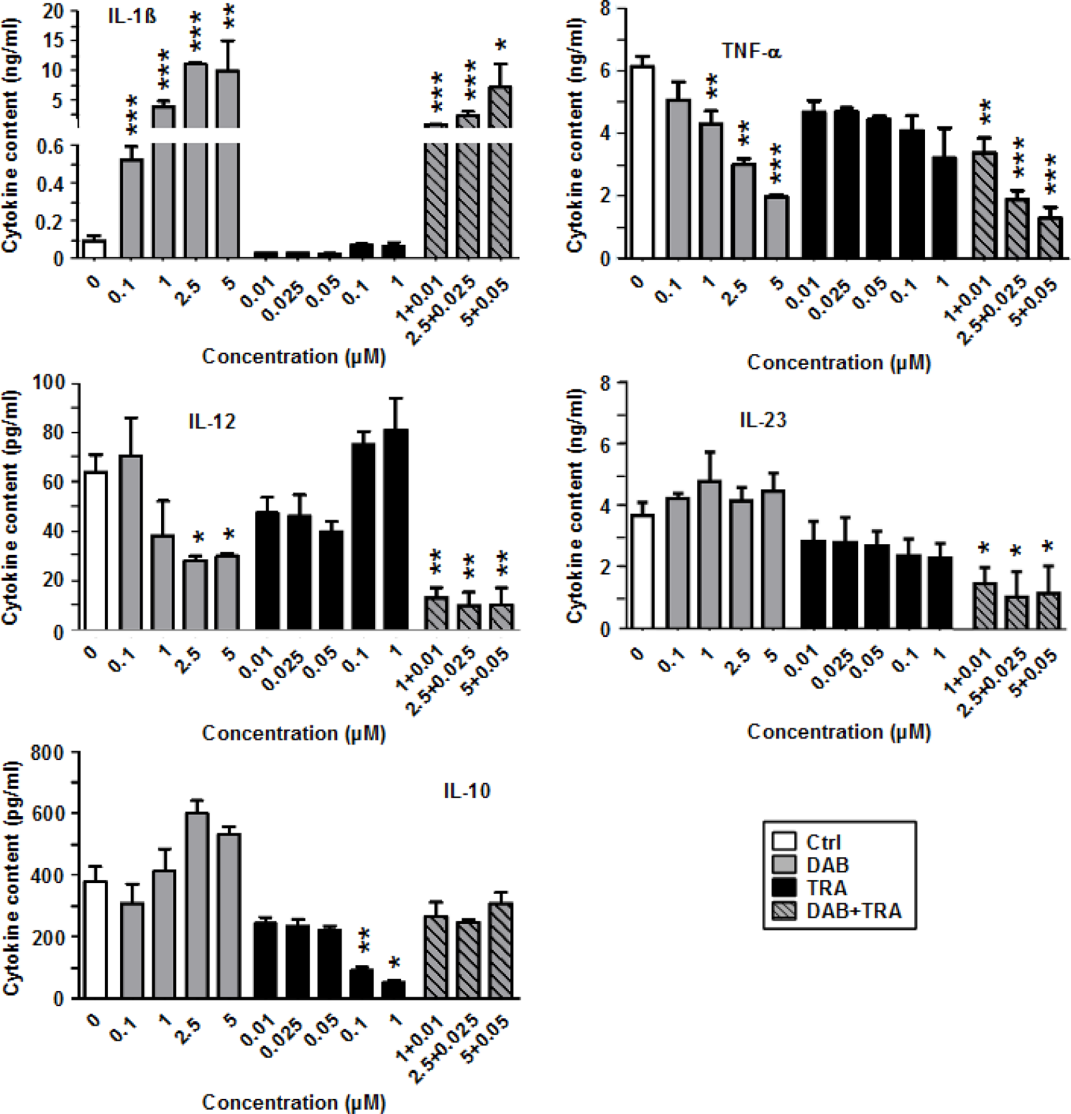
DAB strongly enhances IL-1β generation by stimulated BMDC, but diminishes production of other proinflammatory cytokines BMDC were stimulated with LPS (1 µg/ml). Aliquots were treated in addition with DAB and TRA either alone or in combination at the concentrations indicated. DMSO (1‰) served as a solvent control (Ctrl). After 24 h, supernatants were harvested for cytokine detection by CBA. Data represent mean ± SEM of 3–4 independent experiments each. Statistical differences: ^*^versus control (Ctrl). ^*^p < 0.05, ^**^p < 0.01, ^***^p < 0.001.

In addition, the LPS-induced expression pattern of proinflammatory cytokines (IL-1β, TNF-α, IL-12, IL-23) and of anti-inflammatory IL-10 was altered in response to treatment with DAB and TRA as depicted in Figure 2. Again, DAB strongly upregulated IL-1β secretion in a dose-dependent manner by ∼100-fold as compared with the control. TRA alone exerted no major effect on IL-1β. However, TNF-α production was inhibited by DAB in combination with TRA in a dose-dependent manner. DAB also dose-dependently inhibited IL-12 and IL-23 production which was further diminished when TRA was coapplied. In contrast, DAB moderately elevated IL-10 levels at higher doses (2.5 µM, 5 µM). TRA attenuated IL-10 production when applied alone, and counteracted the moderate stimulatory effect of DAB on IL-10 when coapplied.

These observations indicate that DAB besides its stimulatory effects on the BMDC surface marker phenotype also strongly altered the cytokine pattern of DC, with potent induction of IL-1β and suppression of IL-12, IL-23 and TNF-α production.

### VEM exerts no stimulatory effect on BMDC, but COB elevates MHCII expression

**Figure 3:**
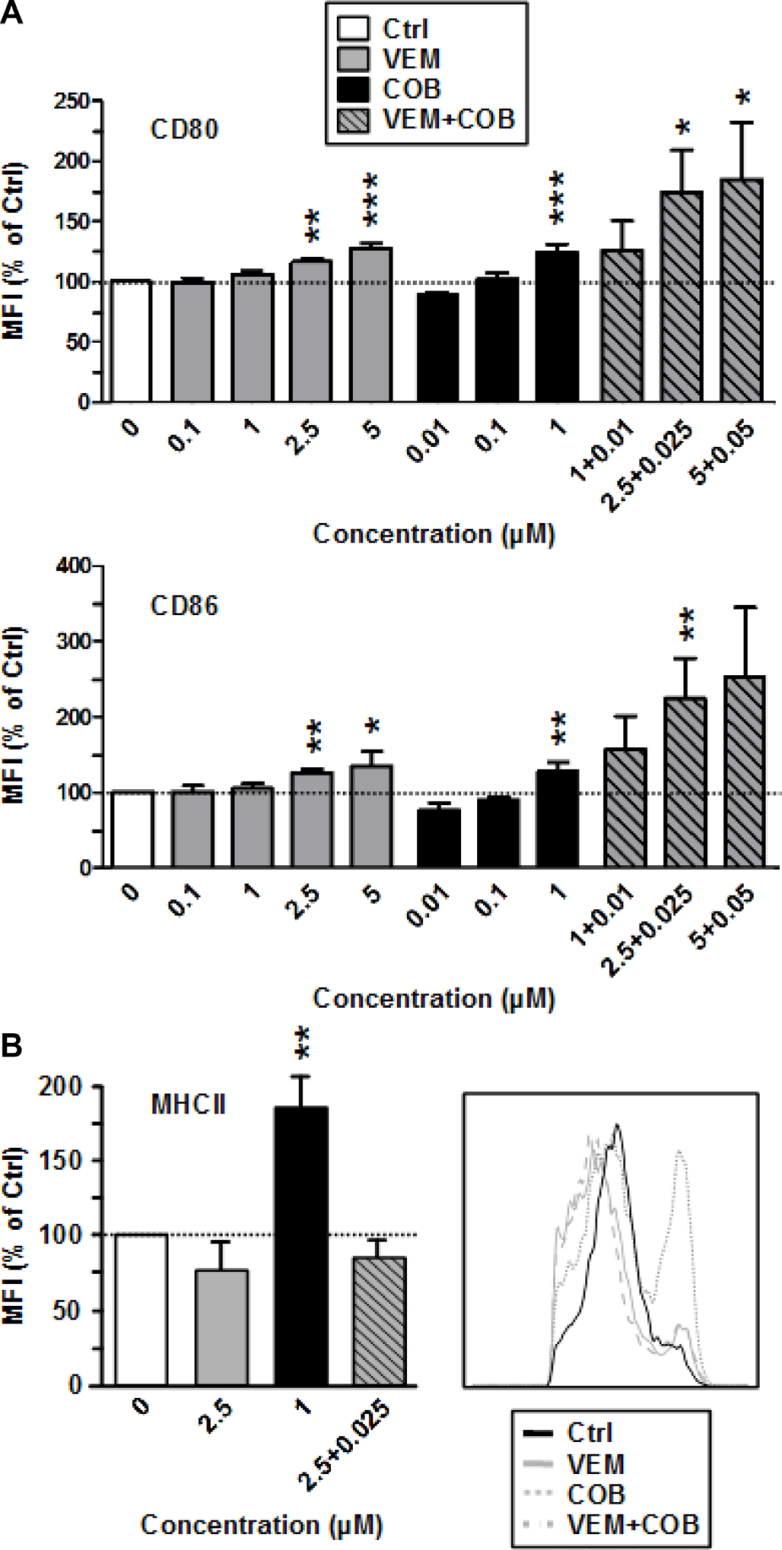
The MEK inhibitor Cobimetinib enhances expression of costimulators (CD80, CD86) by BMDC when coapplied with VEM, and elevates MHCII expression when applied alone VEM and Cobimetinib (COB) were applied alone or in combination at the concentrations indicated to unstimulated BMDC. DMSO (1‰) served as a solvent control (Ctrl). After 24 h, expression of (**A**) CD80, CD86, and (**B**) of MHCII by CD11c^+^ BMDC was assassed by flow cytometry. (A and B, *right panel*) Data show the MFI of the according surface marker on pre-gated CD11c^+^ BMDC, normalized to the expression in Ctrl, arbitrarily set to 100% in each experiment. Data represent the mean ± SEM of 3–4 independent experiments each. (B, *right panel*) Histogram of MHCII expression by CD11c^+^ BMDC after differential treatment is representative of 3 independent experiments each. (A, B) Statistical differences: ^*^versus control (Ctrl). ^*^p < 0.05, ^**^p < 0.01, ^***^p < 0.001.

Next we asked whether the profound effects of DAB and TRA on the immuno-phenotype of DC were agent-specific. To this end, we also included the clinically applied BRAF^V600E^ inhibitor VEM and the coadministerd MEK1/2 inhibitor COB in our analysis. In contrast to DAB, VEM had no effect on the expression of costimulatory surface receptors CD80 and CD86 of BMDC (Figure 3A) as well as the surface level of MHCII (Figure 3B). In agreement, VEM at non-toxic concentrations (Supplementary Figure 2A) yielded no effect on CD86 expression by splenic APC (Supplementary Figure 2B). The MEK1/2 inhibitor COB which is coapplied with VEM in therapy had no effect on the expression of costimulatory surface receptors CD80 and CD86 (Figure 3A), but at high concentration (1 µM) elevated their MHCII expression. However, coapplication of VEM (2.5 µM) and COB (0.025 µM COB) at intermediate doses resulted in enhanced surface levels of both costimulators (CD80, CD86), while MHCII expression remained unaffected. VEM and COB had no effect on the expression of MHCII and costimulators of LPS-stimulated BMDC (not shown).

These results suggest that DAB-induced enhancement of costimulator expression by BMDC is a substance-specific, rather than a drug class-specific off target effect of BRAF^V600E^ inhibitors.

### VEM enhances IL-1β release by LPS-stimulated DC, completely blocked by coapplied COB

**Figure 4:**
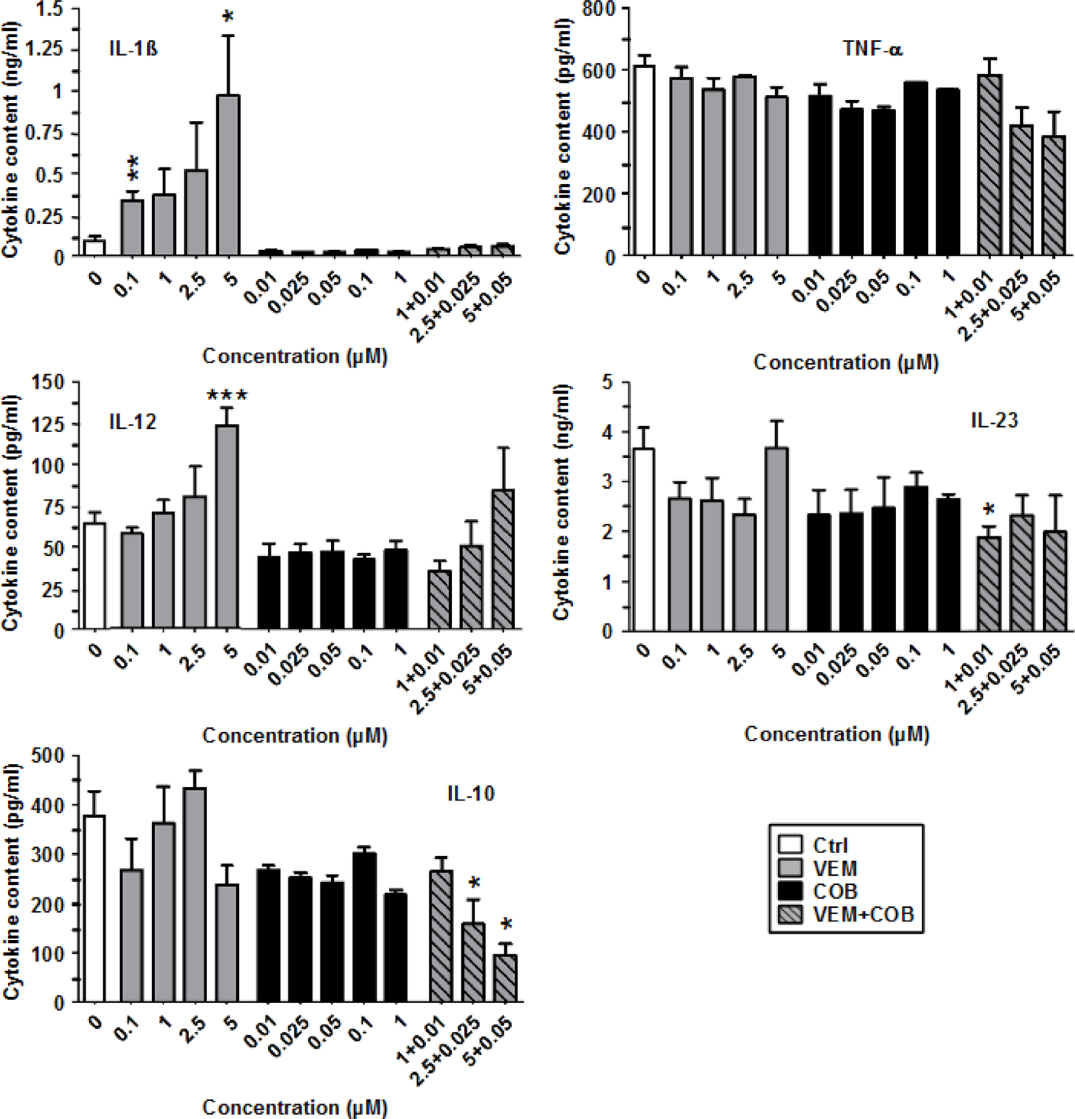
VEM elevates IL-1β production of LPS-stimulated BMDC, abrogated by cotreatment with COB BMDC were stimulated with LPS (1 µg/ml). Aliquots were treated in addition with VEM and COB either alone or in combination at the concentrations indicated. DMSO (1‰) served as a solvent control (Ctrl). After 24 h, supernatants were harvested for cytokine detection by CBA. Data represent mean ± SEM of 3–4 independent experiments each. Statistical differences: ^*^versus control (Ctrl). ^*^p < 0.05, ^**^p < 0.01, ^***^p < 0.001.

Similar to DAB, VEM enhanced IL-1β secretion by LPS-stimulated BMDC in a dose-dependent manner (Figure 4). However, on an equimolar base DAB induced at least 10-fold more IL-1β than VEM. Furthermore, while TRA did not interfere with DAB-dependent IL-1β induction, COB completely abrogated VEM-dependent IL-1β upregulation. With regard to other cytokines, TNF-α and IL-23 were not strongly affected by VEM and COB, in contrast to the inhibitory effects of DAB and TRA on both cytokines. Similarly, while DAB inhibited IL-12, VEM at high concentration (5 µM) enhanced IL-12 levels, largely abrogated upon coapplication of COB (0.05 µM). Similar to DAB, VEM and COB attenuated IL-10 levels, further diminished by coappliction of VEM and COB in a dose-dependent manner.

These results indicate that BRAF^V600E^ inhibitors as a common off target effect elevate IL-1β production, but otherwise qualitatively differ in their effects on other components of the DC immunophenotype.

### VEM and DAB enhance IL-1β expression on mRNA level and via inflammasome activation

As outlined above, of all cytokines monitored, only IL-1β secretion was commonly upregulated by both BRAF^V600E^ inhibitors in LPS-stimulated BMDC, albeit at much higher extent by DAB than by VEM. In addition, DAB (Supplementary Figure 3) but not VEM (not shown) also enhanced IL-1β secretion in unstimulated BMDC. Similar findings were observed for splenic CD11c^+^ DC as assessed by intracellular staining of IL-1β (Supplementary Figure 4, upper panel). However, in this DC population also VEM induced IL-1β producing cells, at similar extent as observed for DAB. CD68^+^ macrophages in spleen showed no major upregulation of the frequency of IL-1β producing cells in response to DAB and VEM (Supplementary Figure 4, lower panel). In contrast, VEM and DAB had no IL-1β promoting effect on splenic CD19^+^ B cells (not shown).

**Figure 5:**
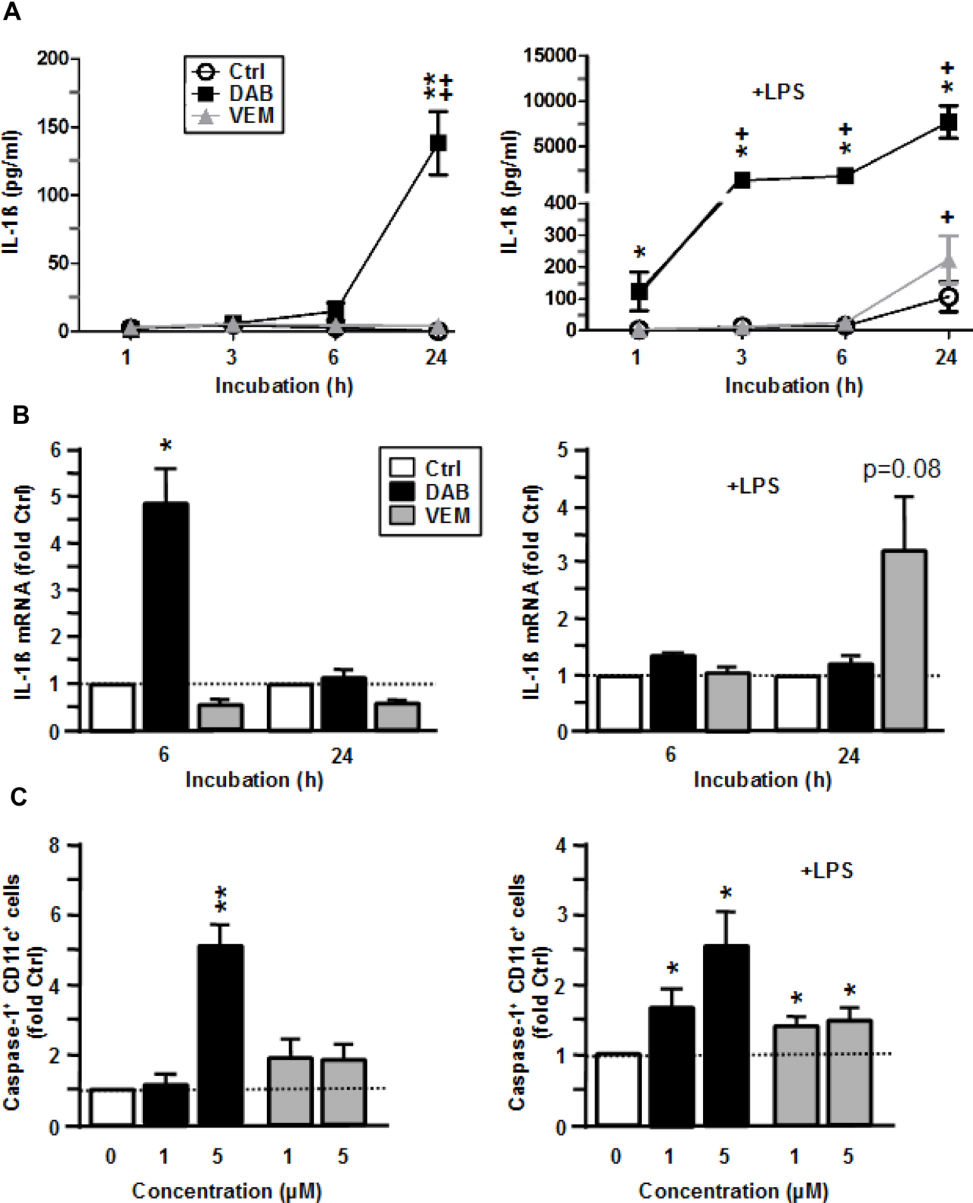
VEM and DAB enhance IL-1β expression on transcriptional level and by inflammasome activation BMDC were treated with VEM and DAB (each 5 µM), and aliquots were cotreated with LPS (1 µg/ml). DMSO (1‰) served as a solvent control (Ctrl). At the indicated time points aliquots were harvested, and (**A**) IL-1β contents were assayed by CBA, and (**B**) IL-1β mRNA levels were assassed by QPCR, and were normalized to expression in the corresponding control. (**C**) After 6 h of coincubation, BMDC were incubated with cell permeant FLICA reagent specifically cleaved to a fluorphoric product by active Caspase-1. BMDCs treated with ATP (5 mM) served as a positive control. Frequencies of Caspase-1 positive cells were assessed by flow cytometry, and values were normalized to the corresponding control. (A–C) Data represent mean ± SEM of 3–4 independent experiments each. Statistical differences: (A–C) ^*^versus corresponding control (Ctrl), (A) ^+^versus control at 1 h. ^*,+^*p* < 0.05, ^**^,^++^*p* < 0.01.

Due to the immunological importance of IL-1β, we asked by which mechanisms the two BRAF^V600E^ inhibitors enhanced IL-1β secretion in myeloid APC. In unstimulated BMDC, DAB induced IL-1β cytokine secretion after 24 h of treatment (Figure 5A, left panel) which corroborated our previous finding (Supplementary Figure 3). Elevated IL-1β secretion was paralleled by transiently enhanced IL-1β mRNA levels 6 h after DAB application (Figure 5B, left panel). Translation of IL-1β mRNA results in pre IL-1β which requires cleavage to yield mature IL-1β protein. Cleavage of pre IL-1β is mediated by an activated inflammasome complex. Inflammasomes consist of multi-protein complexes which are triggered by various stimuli to activate Caspase-1 which in turn cleaves pre IL-1β. We observed that DAB enhanced inflammasome activation in BMDC as reflected by an elevated frequency of Caspase-1^+^ BMDC monitored 6 h after the onset of treatment (Figure 5C, left panel). On the contrary, VEM alone had no effect on IL-1β mRNA levels (Figure 5B, left panel), and a very moderate effect on Caspase-1 activation only (Figure 5C, left panel).

In case of LPS-stimulated BMDC, DAB cotreatment strongly elevated IL-1β cytokine contents at all time points assessed, whereas coapplication of VEM resulted in higher IL-1β protein levels only at 24 h after onset of treatment (Figure 5A, right panel). While DAB exerted no effect on IL-1β mRNA levels in LPS-stimulated BMDC, VEM enhanced IL-1β mRNA contents at 24 h after coapplication (Figure 5B, left panel). DAB-dependently upregulated IL-1β cytokine contents were associated with increased Caspase-1 activation, while VEM evoked only a moderate effect in this regard (Figure 5C, right panel).

Altogether, VEM and DAB elevated IL-1β secretion in a differential manner: While DAB acted on transcriptional level only in case of unstimulated BMDC, and stimulated inflammasome activity in BMDC at either state of activation, VEM elevated IL-1β mRNA levels in LPS-stimulated BMDC only and yielded moderate inflammasome activation.

### DAB and VEM act via NLRC4/caspases-1, and DAB also via caspase-8 to elevate IL-1β release

**Figure 6:**
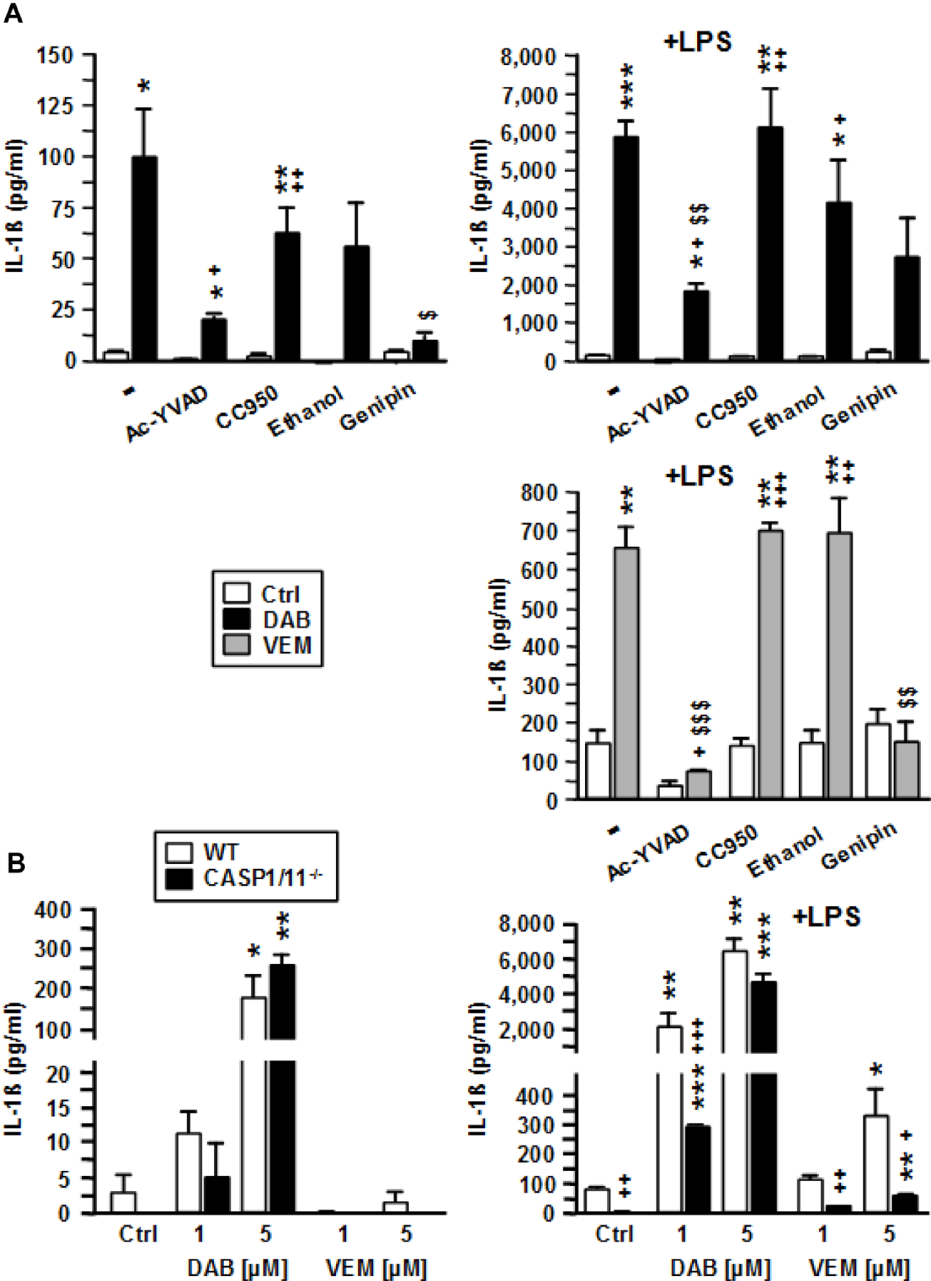
VEM and DAB activate NLRC4/Caspase-1 to induce IL-1β release, but DAB at higher concentration also acts in a Caspase-1/11-independent manner (**A**) BMDC were treated with pharmacological inhibitors of Caspase-1 (Ac-YVAD [50 µM]), NLRP3 (CC950 [100 nM]), NLRP3/AIM2 (ethanol [0.5‰]), and NLRP3/NLRC4 (genipin [50 µM]) at non-toxic concentrations. After 45 min, VEM and DAB (each 2.5 µM) were applied. In case of DAB treatment, aliquots were cotreated with LPS (1 µg/ml). DMSO (1‰) served as a solvent control (-). (**B**) BMDC (wild type [WT], Casp1/11^--^) were treated with VEM and DAB (each 1 µM and 5 µM), and aliquots were cotreated with LPS (1 µg/ml). DMSO (1‰) served as a solvent control (Ctrl). (A, B) On the next day, IL-1β contents of BMDC supernatants were assayed by CBA. Data represent mean ± SEM of 3 (B) or 4 (A) independent experiments each. Statistical differences: ^*^versus corresponding control (Ctrl), ^+^versus WT at same conditon, ^$^versus BRAF^V600E^ inhibitor-treated sample w/o additional inflammasome inhibitor. ^*,+^*p* < 0.05, ^**,++,$$^*p* < 0.01, ^***,+++,$$$^*p* < 0.001.

To elucidate which molecular components of the inflammasome were activated by DAB and VEM, BMDC were pretreated with respective inhibitors prior to application of intermediate doses of either BRAF^V600E^ inhibitor (each 2.5 µM). DAB-mediated induction of IL-1β was strongly attenuated in response to inhibition of Caspase-1 with Ac-YVAD, and upon application of the dual NLRP3/NLRC4 inhibitor genipin, but only moderately affected when using specific NLRP3 (CC950) and dual NLRP3/AIM2 (ethanol) inhibitors (Figure 6A, upper left panel). As previously observed, cotreatment of BMDC with DAB and LPS resulted in much stronger upregulation of IL-1β as compared with application of DAB or LPS alone. Under these conditions, only inhibition of Caspase-1 and NLRC4 markedly attenuated IL-1β levels (Figure 6A, upper right panel). VEM-induced IL-1β upregulation was abolished upon inhibition of Caspase-1, and in the presence of genipin (Figure 6A, lower panel). Altogether, these findings suggest that both BRAF^V600E^ inhibitors act via canonical NLRC4/Caspase-1 inflammasome activation to elevate IL-β secretion.

To confirm the importance of Caspase-1 for BRAF^V600E^ inhibitor induced IL-1β release, we employed Caspase-1/11^-/-^ BMDC. Of note, DAB alone at high concentration (5 µM) induced IL-1β in BMDC irrespective of Caspase-1/11 expression (Figure 6B, left panel). When coapplied with LPS, DAB at lower dose (1 µM) induced IL-1β release also in Caspase-1/11^-/-^ BMDC, but at markedly lower levels as compared with the corresponding wild type (WT) control (Figure 6B, right panel). However, when DAB was coapplied with LPS at higher dose (5 µM) IL-1β levels were comparable in supernatants of both BMDC populations. In contrast, VEM induced IL-1β only when coapplied with LPS. These results suggested that while VEM critically depends on Caspase-1 activation, DAB at higher concentration also acted in a Caspase-1 independent manner. Pharmacological inhibiton of Caspase-8 strongly inhibited DAB-induced IL-1β secretion (DAB alone: by 56%, DAB+LPS: by 88%; not shown).

Altogether, these results indicate that DAB and VEM commonly act via NLRC4/Caspase-1 inflammasome activation to promote maturation of pre IL-1β. However, DAB but not VEM at high dose is able to activate Caspase-8 for IL-1ß maturation as an alternative mechanism.

### BRAF^V600E^ inhibitors modulate the immuno-phenotype of human monocyte-derived (MO)DC, and DAB induces IL-1β in human primary DC

Finally, we asked for effects of BRAF^V600E^ inhibitors on human DC. For this, we differentiated MODC derived from progenitors of healthy volunteers. In contrast to our findings in mouse, VEM but not DAB yielded enhanced costimulator expression (Supplementary Figure 5A). However, in accordance with BMDC, DAB applied for 6 h upregulated IL-1β mRNA expression (Supplementary Figure 5B), and both BRAF^V600E^ inhibitors moderately upregulated Caspase-1 activity when applied for 6 h (Supplementary Figure 5C). Unfortunately, we detected no elevated IL-1β in supernatants of BRAFi-treated MODC (data not shown). However, when testing effects of BRAFi on primary human DC isolated from PBMC we observed an increase in IL-1ß levels in most samples after treatment with DAB (Supplementary Figure 5D), but not for VEM (not shown). This finding is in agreement with the observation of marked induction of IL-1ß mRNA and Caspase-1 by DAB in unstimulated MODC. Altogether, these findings show that BRAF^V600E^ inhibitors exert yet undescribed immunomodulatory effects on APC in a DC population- and species-dependent manner.

## DISCUSSION

The BRAF^V600E^ inhibitors VEM and DAB are one treatment of choice for BRAF^V600E^ metastatic melanoma [18]. Due to frequent occurence of tumor resistance towards treatment both BRAF^V600E^ inhibitors are coapplied with according MEK inhibitors [10, 11]. These combinations are more effective to abolish ERK hyperactivity in melanoma and exert sustained activity. Tumor cell death is induced by ERK inhibition, but immuno-relevant mechanisms like enhanced antigen presentation by tumor cells as induced by BRAF^V600E^ inhibitors [13], and paradoxical ERK activation in effector T cells as induced by MEK inhibitors [16] may also contribute to therapeutic effects.

In light of the role of DC for the induction and polarization of adaptive anti-tumor responses [17] here we asked for potential off target effects of BRAF^V600E^ inhibitors and for the immuno-modulatory role of coapplied MEK inhibitors at therapeutically relevant doses.

We demonstrate that DAB alone partially activates mouse splenic APC populations and BMDC as reflected by elevated expression of MHCII and costimulators (CD80, CD86), while VEM yielded no such effect. ERK signaling was reported to maintain DC in an immature state [19]. In agreement, both MEK inhibitors (COB, TRA) induced upregulation of MHCII by BMDC, but had no effect on costimulator expression. However, coapplication of VEM and COB yielded stimulatory effects on CD80 and CD86 expression. Similarly, combined treatment with DAB and TRA at the lowest concentrations tested induced profound elevation of both costimulators. Somewhat different to our findings in mouse, VEM alone upregulated costimulator expression by human MODC while DAB exerted no stimulatory effect. In previous studies VEM [20] and DAB [21] were reported to yield no immuno-modulatory effects on human MODC. Interestingly, Tel and coworkers [22] showed that VEM, but not DAB interfered with R848-induced upregulation of surface activation markers and cytokines by human primary DC. However, the inhibitiory effect of VEM on DC activation was largely prevented when applying VEM to total PBMC.

In agreement with the partially stimulatory activity of TRA on mouse BMDC, Vella and coworkers demonstrated a stimulatory effect of this MEK inhibitor on MODC as well, while the opposite was reported by Ott *et al.* [20] which may be attributed to methodological differences in DC differentiation.

Unexpected DC stimulatory properties have been reported for other anti-tumor drugs as well. In this regard, we have previously demonstrated that topotecan [23] and the heat shock protein 90 inhibitor geldanamycin [24] stimulated MODC which was associated with NF-kB activation. Further studies are required to identify the molecular targets of BRAF^V600E^ inhibitors in DC which contribute to cellular stimulation. When treated with LPS as a very potent DC stimulator, BRAF^V600E^ inhibitors and MEK inhibitors exerted no additional modulatory effect on the expression of DC activation markers (data not shown). Similar observations were obtained in our aforementioned studies on the DC-stimulatory effects of chemotherapeutics. Our finding of DAB-mediated upregulation of MHCII and costimulators by DC suggests that DAB as an off-target effect stimulates NF-kB activation via yet unknown molecular targets.

In our study, the BRAF and MEK inhibitors tested altered the cytokine pattern of LPS-stimulated BMDC. On an equimolar base DAB and TRA mediated stronger inhibitory effects than VEM and COB in case of TNF-α, IL-12 and IL-23. In contrast to these inhibitory effects, IL-1β was upregulated by both BRAF^V600E^ inhibitors, with DAB being much more potent on an equimolar base than VEM. Besides, while COB abolished VEM-induced IL-1β upregulation, TRA exerted no inhibitory effect on DAB-dependent IL-1β upregulation. Both BRAF^V600E^ inhibitors also enhanced IL-1β secretion by splenic DC and to minor extent by macrophages, but not by B cells.

In several studies chemotherapeutics including doxorubicin, daunorubicin [25] and vincristine [26] were demonstrated to elevate IL-1β release by DC and macrophages by triggering the NLRP3 inflammasome, and subsequent Caspase-1 activation. These drugs act on primed myeloid APC characterized by elevated IL-1β transcription and pre IL-1β production. We show that even DAB alone elevated IL-1β secretion by unstimulated BMDC via transcriptional upregulation of IL-1β mRNA, and inflammasome activation, which was also observed in case of human MODC. In contrast, VEM elevated IL-1β levels only in LPS-stimulated BMDC primarily by enhancing IL-1β mRNA expression at later time points. However, in human MODC also VEM exerted moderate Caspase-1 activation. Altogether, these results suggest that BRAF^V600E^ inhibitors act at both levels of IL-1β regulation in a species- and DC population-dependent manner.

In clinical practice, drug side effects to BRAF^V600E^ inhibitors differ markedly between patients: whereas some suffer from pronounced rash towards VEM or fever attacks in response to DAB, other patients show no such side effects. Thus, there appear to exist genetic polymorphisms that control these immunomodulatory effects of BRAF^V600E^ inhibitors in humans. This assumption is confirmed by our finding that a fraction of human primary DC sampels did not responded to DAB treatment with regard to elevated IL-1β production.

As mentioned above, several chemotherapeutics were demonstrated to activate the NLRP3 inflammasome shown to sense various pathogen-derived and endogenous danger signals [25]. However, we show that the BRAF^V600E^ inhibitors tested elevated IL-1β in a NLRP3-independent manner, but via NLRC4. So far, flagellin has been identified as the major inducer of the NLRC4 inflammasome [27]. We also show that VEM requires Caspase-1 to cleave pre IL-1β, while DAB at higher concentrations induced IL-1β independent of Caspase-1. Pharmacological inhibition of Caspase-8 partially inhibited DAB-mediated IL-1β induction. Besides activation of the NLRP3/Caspase-1 axis, more recently doxorubicin and staurosporine were also found to promote IL-1β maturation in a Caspase-1 independent manner via a FADD/Caspase-8 complex termed riptoptosome [28]. Further studies are necessary to clarify which proteins are targeted by DAB to facilitate IL-1β release.

IL-1 has early been attributed anti-apoptotic and stimulatory effects on DC [29]. In agreement, IL-1β was demonstrated to play an essential role in the induction of adaptive anti-tumor immune responses [30]. In chemically induced colon cancer models mice with a defective inflammasome showed stronger tumor induction and progression than WT mice suggesting a protective role of the inflammasome [31]. In contrast, in other tumor models including melanoma, inflammasome activity supported tumor growth [32]. In line, many human tumors including advanced melanomas are characterized by high level IL-1β production due to both elevated pre IL-1β expression and enhanced Caspase-1 activity [33]. Several studies have revealed different tumor-promoting effects of IL-1β. In an autocrine manner, tumor-derived IL-1β stimulated melanoma cell proliferation [34]. In addition, tumor-derived IL-1β was shown to be essential for conversion of fibroblasts into cancer-associated fibroblasts [35], which expressed the immuno-suppressive receptors PD-L1 and PD-L2 [14]. IL-1β generated by tumor cells and by tumor-associated macrophages in an autocrine manner induced CCL2 secretion which in turn attracted macrophages [30]. Furthermore, transcriptional reprogramming of tumor-infiltrating myeloid cells was mediated in part by IL-1β induced NF-kB activation [36]. Besides, tumor neoangiogenesis was shown to be promoted by IL-1β induced COX-2 [37]. Above, IL-1 was shown to stimulate the expression of endothelial adhesion molecules that may favor interaction with spreading tumor cells, and hence metastasis [38].

While inflammasome activity in tumor cells enabled the aforementioned tumor-promoting effects, immune cells that produce IL-1β exert stronger tumor-suppressive function [39]. In agreement, anti-tumor chemotherapeutics are most efficient when they induce immunogenic tumor cell death, accompanied by IL-1β induction in immune cells [32]. The latter is initiated by the release of endogenous danger signals like ATP derived from mitochondria of necrotic tumor cells [39] which stimulates the NLRP3 inflammasome of myeloid APC by binding to purinergic receptors [40]. In an autocrine manner, IL-1β stimulates APC via activation of NF-kB [41]. Moreover, APC-derived IL-1β promotes T cell-driven anti-tumor responses by favouring Tc1 [42] and Th1/Th17 polarization [43], and by activating γδ T cells [44]. In agreement with these beneficial effects of IL-1β generated by APC, mice upon blockade of IL-1 signaling [45] do not respond to chemotherapy with immunogenic cell death inducers like anthracyclines.

In metastatic melanoma the BRAF^V600E^ mutation was reported to elevate production of IL-1α and IL-1β, abrogated after treatment with VEM [14]. We show here that BRAF^V600E^ inhibitors promote IL-1β secretion in DC via the NLRC4 inflammasome, and in case of DAB also in a Caspase-1 independent manner. Therefore, BRAF^V600E^ inhibitors in a cell type- or microenvironment-specific manner inhibit (tumor) and promote (APC) IL-1β secretion. On the contrary, currently used chemotherapeutics activate the NLRP3 inflammasome at differential extent in a more unspecific manner and thereby may cause side effects such as cardiotoxicity (anthracyclines) [25], intestinal mucositis (5-fluoruracil) [46], or pulmonary fibrosis (bleomycin) [47]. To prevent these IL-1β related cytotoxic effects as well as adverse effects of IL-1β within the tumor microenvironment (see above), administration of IL-1β inhibitors has been suggested [32]. However, systemic IL-1β inhibition may compromise anti-tumor immune responses that depend on immunogenic cell death, and immune responses against infections [48].

Further studies are required to elucidate the cell type specificity of BRAF^V600E^ inhibitor induced IL-1β secretion. In case of APC-restricted inflammasome activation these agents may be well suited to exploit the beneficial effects of APC-associated IL-1β for tumor therapy.

## MATERIALS AND METHODS

### Reagents

VEM, DAB, COB and TRA were obtained from Selleck Chemicals (Houston, TX, USA). Ac-YVAD-cmk, CC950, and Genipin were purchased from Tocris (Bristol, UK), and ethanol was obtained from Roth (Karlsruhe, Germany).

### Cells

Female C57BL/6J mice were obtained from Janiver Labs (Saint Berthevin Cedex, France) and were maintained in the Translational Animal Research Center of the University Medical Center Mainz under pathogen-free conditions on a standard diet. The recommendations of the Guide for the Care and Use of Laboratory Animals by the National Institutes of Health were followed.

Spleen cells were isolated from spleens of 6–12 week old mice, using a 40 µM cell strainer (Greiner Bio-One, Frickenhausen, Germany) to generate a single cell suspension in culture medium (IMDM with 5% Fetal Bovine Serum, 2 mM L-glutamine, 100 U/ml penicillin, 100 µg/ml streptomycin [all from Sigma-Aldrich, Deisenhofen, Germany], and 50 µM ß-mercaptoethanol [Roth, Karlsruhe, Germany]).

Bone marrow (BM)-derived DC (BMDC) were differentiated from progenitors of 6–12 week old mice as described [49]. To this end, BM cells (2 × 10^5^/ml) were seeded either in 12 well plates (1 ml) or in 10 cm bacterial dishes (10 ml) (both from Greiner Bio-One) in culture medium (see above) supplemented with 10 ng/ml recombinant murine GM-CSF (R&D Systems, Wiesbaden, Germany). Media was replenished on days 3 and 6 of culture. Aliquots of non-adherent and loosely adherent immature BMDC were harvested on days 7 or 8 of culture for subsequent experiments. Where indicated, BRAF^V600E^ and MEK1/2 inhibitors were coapplied. In all other cases of BMDC treatment with different agents, inflammasome inhibitors were applied first, and BRAF^V600E^ inhibitors, and LPS (1 µg/ml, Merck Millipore, Billerica, MA, USA) were administered sequentially at temporal distances of 1 h. DMSO (1‰) served as solvent control. One day later samples were analysed.

### Surface marker expression

BMDC cultures (12 well plates) and freshly isolated spleen cells (5 × 10^5^/ml) seeded in 12 well plates (1 ml) were treated with inhibitors and LPS. One day later, cells were harvested and washed in staining buffer (PBS/2% FCS). Cells were incubated with rat anti-mouse CD16/CD32 antibody (clone 2.4G2) for 15 min at room temperature. Afterwards, spleen cells were incubated with (APC)-conjugated anti-CD11c (clone N418), PE-conjugated anti-CD68 (clone FA-11), FITC-conjugated anti-CD19 (clone 1D3) and PE-Cy7-labeled anti-CD86 (GL1) antibodies. BMDC were incubated with APC-conjugated anti-CD11c, eFluor450-conjugated anti-MHC class II (clone M5/114.15.2), FITC-conjugated anti-CD80 (16-10A1), and PE-Cy7-labeled anti-CD86 antibodies. All antibodies were purchased from Biolegend or affymetrix/eBioscience (both San Diego, CA, USA). Samples were analyzed using a BD FACS CANTO II flow cytometer (BD Biosciences, San Jose, CA, USA). Data were analyzed using FlowJo software (FLOWJO, Ashland, OR, USA). The gating strategy is depicted in Supplementary Figure 6.

### Caspase-1 activity

To detect activated Caspase-1 in BMDC, the Flica 660 Caspase-1 Assay Kit (ImmunoChemicals, Bloomington, USA) was used following the manufacturer’s instructions. For this, BMDC (12 well plates) were treated with the indicated agents for 6 h prior to analysis, and assayed by flow cytometry (see above)

### Cytokine detection

Cytokine contents of BMDC supernatants were quantified by Cytometric bead array (BD Bioscience). For this, BMDC (10 cm dishes) were harvested and were reseeded in cell-culture treated 24 well plates (10^6^/ml). BMDC were differentially treated, and supernatants were collected on the next day and analyzed using the mouse CBA flex sets following the manufacturer´s instructions.

### Real-time PCR analysis

Total RNA was isolated from BMDC using the RNeasy MiniPlus kit (Qiagen, Hilden, Germany), and reverse-transcribed by employing the iScript kit (Bio-Rad, Munich, Germany) as recommended by the manufacturer. Primer pairs used to detect expression of IL-1β and the house-keeping gene ubiquitin C were obtained from eurofins MWG Synthesis (Ebersberg, Germany). Primer sequences and data analysis have been described [49].

### Statistical analysis

Data are presented as means ± SEM of the values. Data were analyzed for statistically significant differences by applying one-way ANOVA followed by Tukey´s test for post hoc analysis using SigmaPlot 12.3 (Systat Software, San Jose, CA).

## SUPPLEMENTARY MATERIALS FIGURES



## Abbreviations

AIM2: absent in melanoma 2
APC: antigen presenting cell
BMDC: bone marrow-derived dendritic cell
BRAF: v-Raf murine sarcoma viral oncogene homolog B
CD: cluster of differentiation
COB: Cobimetinib
COX-2: Cyclooxygenase-2
DAB: Dabrafenib
DC: dendritic cell
ERK: extracellular signal regulated kinase
FADD: Fas (tumor necrosis factor receptor superfamily 6)-associated via death
IL: interleukin
LPS: lipopolysaccharid
MEK: mitogen-activated protein kinase/ERK kinase
MODC: monocyte-derived DC
NF-kB: nuclear factor - kappa-light-chain-enhancer of activated B-cells
NLRC4: ‘nucleotide-binding domain, leucine-rich repeat’ family, CARD domain containing 4
NLRP3: ‘nucleotide-binding domain, leucine-rich repeat’ family, pyrin domain containing 3
PD-L1: programmed death-ligand 1
PD-L2: Programmed death-ligand 2
Tc1: cytotoxic T cell type 1
Th1: T helper cell type 1
Th17: Th type 17
TNF-α: tumor necrosis factor alpha
TRA: Trametinib
VEM: Vemurafenib
