# Supplementary material for: BRAF inhibitors stimulate inflammasome activation and interleukin 1 beta production in dendritic cells
Source: Oncotarget. 2018 Jun 19;9(47):28294–308. doi: 10.18632/oncotarget.25511 (PMC6033361; doi:10.18632/oncotarget.25511)
Supplement: Supplementary file 1 [file oncotarget-09-28294-s001.pdf]

BRAF inhibitors stimulate inflammasome activation and interleukin 1 beta production in dendritic cells

SUPPLEMENTARY MATERIALS

MATERIALS AND METHODS

Cytotoxicity

A non-radioactive cell proliferation assay (Promega, Mannheim, Germany) was used to assess metabolic activity of cells as recommended by the manufacturer. For this, BMDC (2.5×10^5/100 µl) were seeded in a 96 well plate and treated with agents as indicated in triplicates. After 24 h incubation period, 20 µl of Dye Solution were added to each well, and after another 4 h 100 µl of Solubilization Solution were applied. After solubilization formazan product absorbance was measured in an ELISA reader at 570 nm.

Viability of differentially treated BMDC was assessed after incubation (15 min) with AF647-labeled Annexin V (Biolegend, San Diego, CA) to assess surface-exposed phosphatidylserine of apoptotic cells, and with 7-AAD (Biolegend) that enters necrotic/late apoptotic cells to intercalate chromosomal DNA at room temperature. Samples were assayed within 1 h by flow cytometry.

Generation of human monocyte-derived DC (MODC) and isolation of primary human DC

PBMC were isolated from buffy coats (provided by the Transfusion Service of the University Medical Center Mainz, Germany) using Biocoll separating solution (density 1,077 g/ml; Merck, Darmstadt, Germany). Cells were washed three times with PBS + 0,5 M EDTA and diluted 9×10^6 cells/ml in RPMI 1640 medium plus 1.5% heat-inactivated autologous plasma. Monocytes were enriched by the plastic adherence method [1]. After 45 min incubation at 37° C in a 5% CO_2 incubator non-adherent cells were carefully washed off petri-dishes. The remaining adherent monocytes were cultured in 15 ml *X-VIVO* 15 (Lonza, Basel, Switzerland) with GM-CSF (800 I.U./ml [Sagramostim]; Sanofi, Paris, France) and IL-4 (200 I.U./ml; ImmunoTools, Friesoythe, Germany) for five days. One third of medium was replaced every other day (GM-CSF, 800 I.U./ml; IL-4, 200 I.U./ml). On day 6, two thirds of the MODC cultures were harvested and 10^6 cells/well were seeded into 6 well plates. To induce maturation of MODC, fresh media was supplemented with a maturation cocktail composed of IL-4 (200 IU/ml), GM-CSF (400 IU/ml), IL-6 (1000 IU/ml; ImmunoTools), IL-1β (10 ng/ml; Cellgenix, Freiburg, Germany), PGE_2 (1 µg/ml; Tocris, Bristol, UK) and TNF-a (10 ng/ml; Miltenyi, Bergisch Gladbach, Germany) for 48 h. Aliquots were treated with inhibitors as indicated on day 6 of culture.

Primary human DC were purified from PBMC by immunomagnetic sorting using the blood dendrtiic cells isolation kit II (Miltenyi Biotec, Bergisch Graldbach, Germany). Purity of sorted DC was monitored by assessing the frequencies of contaminating $CD14^+$ monocytes and $CD19^+$ B cells by flow cytometry as recommended by the manufacturer, which was regularily below 3%. DC were resuspended in RPMI160 medium supplemented with 1.5% autologous heat-inactivated plasma (10^6 cells/ml) in wells of 96 well plates (100 µl) for subsequent treatment.

Real-time PCR analysis

Total RNA was isolated from MODC using the peqGold Micro RNA kit (S-Line) (vwr, Darmstadt, Germany) and reverse-transcribed by employing the iScript kit (Bio-Rad, Munich, Germany) or All-in-one cDNA Super Mix (Bimake, Houston, USA) as recommended by the manufacturer. Primer pairs used to detect expression of IL-1β (sense: 5′-GGACAAGCTGAGG AAGATGC-3′, anti-sense: 5′-TCGTTATCCCATGTGT CGAA-3′) and the reference gene b2M (sense: 5′-AT GAGTATGCCTGCCGTGTGA-3′, anti-sense: 5′-GGCA TCTTCAAACCTCCATG-3′) were obtained from eurofins MWG Synthesis (Ebersberg, Germany).Relative gene expression was quantified using delta delta CT method.

Flow cytometry

MODC were stained utilizing unlabeled primary antibodies and fluorochrome-conjugated secondary antibodies. The following unlabeled primary antibodies were used: mouse anti-human CD1a (BD, Franklin Lakes, NJ, USA), mouse anti-human CD14 (Beckman Coulter, Brea, CA, USA), mouse anti-human CD80 (Biolegend, San Diego, CA, USA), mouse anti-human CD86 (Hercules, CA, USA) and rat anti-human MHC II HLA-DR antibody (Thermo Fisher Scientific, Waltham, MA, USA). Secondary antibodies used were R-PE-

conjugated AffiniPure $F(ab')_2$ Fragment Donkey anti-mouse IgG (H+L) (Jackson ImmunoResearch, West Grove, PA, USA) and donkey anti-rat IgG (H+L) $F(ab')_2$ Fragment FITC (Dianova, Hamburg, Germany). Dead cells were excluded by 7-AAD viability staining solution (eBioscience, Waltham, MA, USA). After fixaton with 4 % formaldehyde PBS solution, cells were analyzed on a BD Accuri.

Detection of caspase-1 activity

MODC were harvested on day 6 of culture, and were treated as indicated for 6 h. Caspase-1 activity was detected using the FLICA 660 Caspase-1 Assay Kit (ImmunoChemicals) according to manual, and analyzed on a BD Accuri.

REFERENCE

1. Jonuleit H, Kühn U, Müller G, Steinbrink K, Paragnik L, Schmitt E, Knop J, Enk AH. Pro-inflammatory cytokines and prostaglandins induce maturation of potent immunostimulatory dendritic cells under fetal calf serumfree conditions. Eur J Immunol. 1997; 27:3135–42.

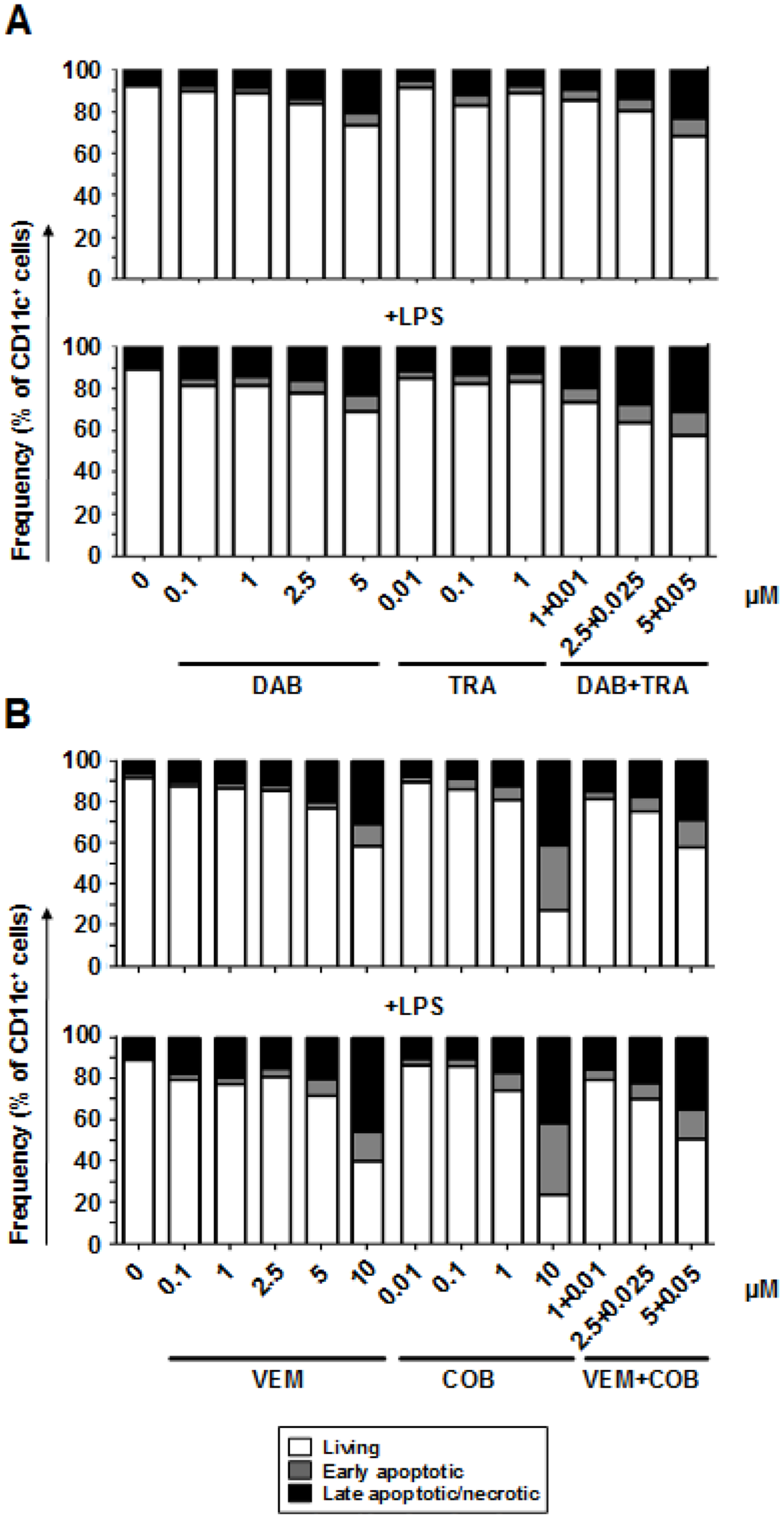

Supplementary Figure 1: Toxicity of BRAF^{V600E} and MEK inhibitors on the viability of BMDC. BMDC (2.5×10^5 cells/ml) were incubated with (**A**) VEM and COB and (**B**) DAB and TRA applied either alone or at corresponding combinations at the concentrations indicated. (A and B, lower panels) Aliquots were cotreated with LPS (100 ng/ml). DMSO (1‰) served as a solvent control. (A, B) After 24 h, BMDC were incubated with Annexin-V and 7-AAD, and analyzed by flow cytometry. Data show the frequencies of living (Annexin-V$^-$7-AAD$^-$), early apoptotic (Annexin V$^+$7-AAD$^-$), and late apoptotic/necrotic cells (Annexin-V$^+$7-AAD$^+$+Annexin-V$^-$7-AAD$^+$). Data represent mean ± SEM of 3-4 independent experiments each.

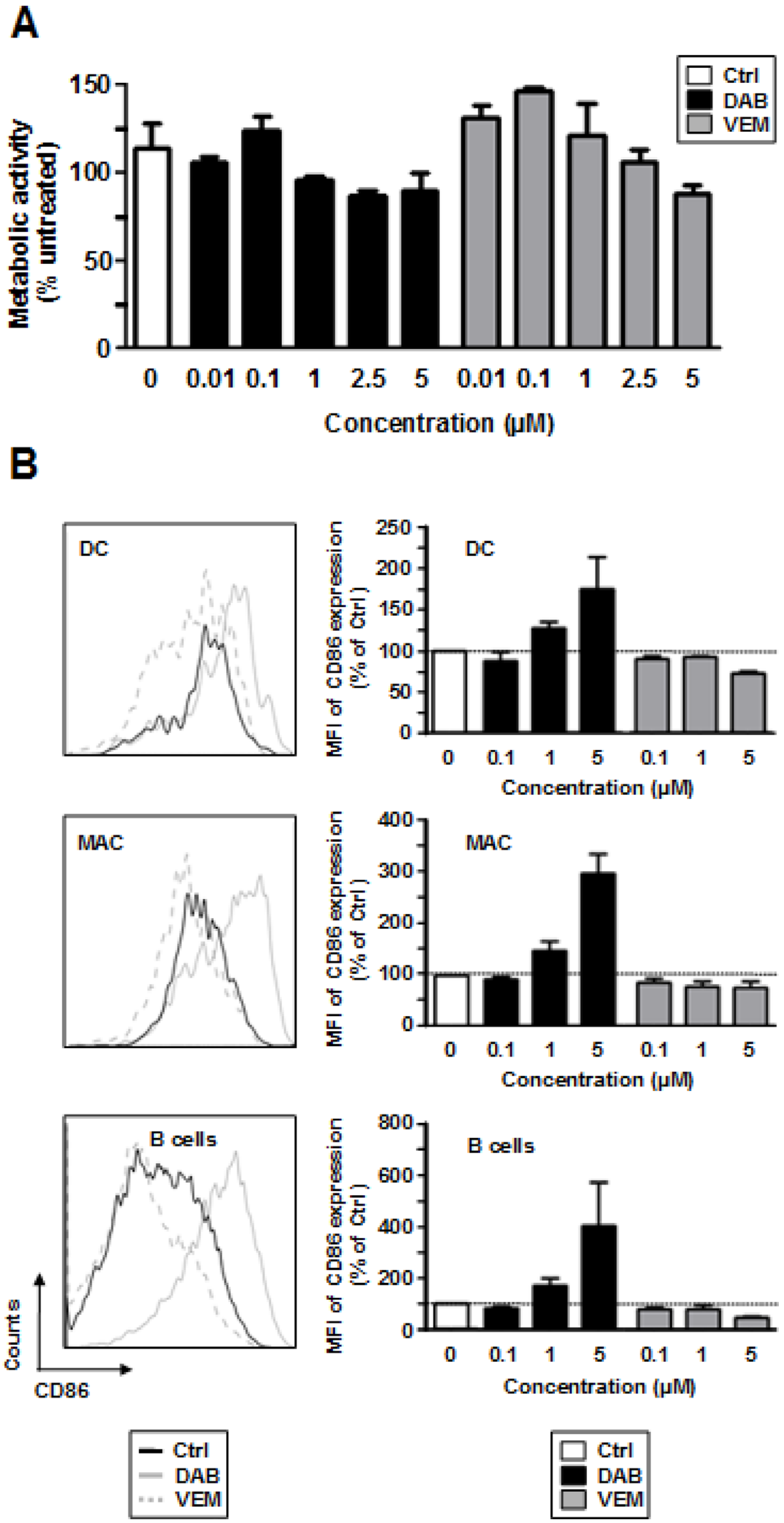

Supplementary Figure 2: The BRAF^{V600E} inhibitor DAB upregulates expression of CD86 by splenic APC populations. Freshly isolated spleen cells (each 10^6) were treated with the BRAF^{V600E} inhibitors VEM and DAB at the concentrations indicated in triplicates. DMSO (1‰) served as a solvent control (Ctrl). (**A**) After 24 h, metabolic activity was assessed by MTT assay. Data show the mean ± SEM of 3 independent experiments. (**B**) Expression of CD86 by DC ($CD11c^+$) macrophages (MAC; $CD68^+$), and B cells ($CD19^+$) was assessed by flow cytometry. Left panels, Histograms of CD86 expression by either cell population are representative of 3 independent experiments each. Right panels, Quantification of CD86 expression by the according APC populations. Data show the mean fluorescence intensity (MFI) of CD86, normalized to the expression in DMSO-treated samples (Ctrl), arbitrarily set 100% in each experiment. Data represent the mean ± SEM of 3 independent experiments each.

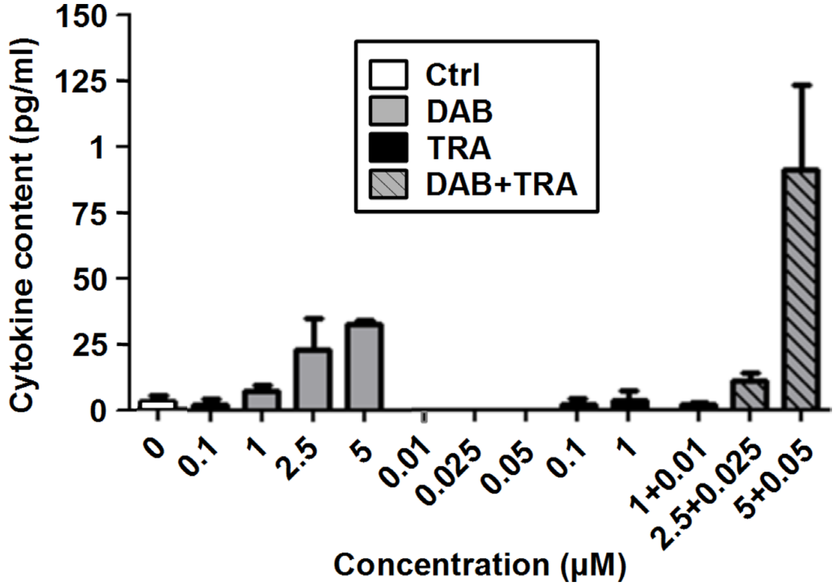

Supplementary Figure 3: DAB induces IL-1ß production in unstimulated BMDC. BMDC were treated with DAB and TRA applied either alone or at corresponding combinations at the concentrations indicated. DMSO (1‰) served as a solvent control. After 24 h, supernatants were harvested for detection of IL-1ß by CBA. Data represent mean ± SEM of 3–4 independent experiments each.

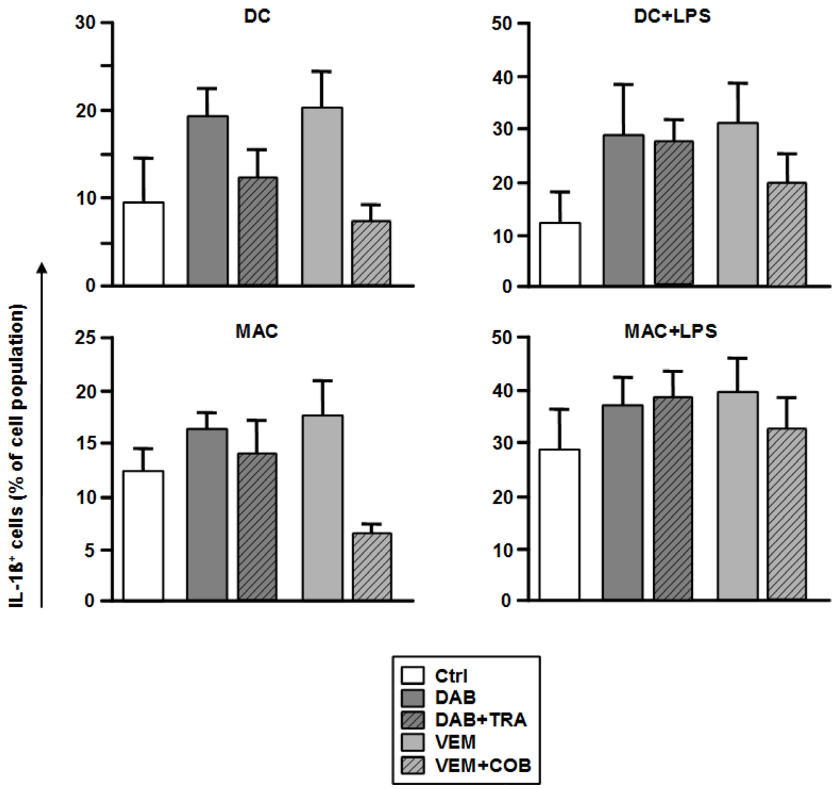

Supplementary Figure 4: VEM and DAB enhance IL-1ß production by splenic DC and monocytes/macrophages. Freshly isolated spleen cells (each 10^6) were stimulated for 24 h with LPS in the presence of DAB at the concentrations indicated. DMSO (1‰) served as a solvent control (Ctrl). Intracellular expression of IL-1ß in splenic DC (CD11c$^+$) and monocytes/macrophages [MAC] (CD11c-F4/80$^+$), and B cells (CD19$^+$) was assessed by flow cytometry. Data show the MFI of IL-1ß of the accordingly pre-gated spleen cell population. Data represent the mean ± SEM of 3 independent experiments each.

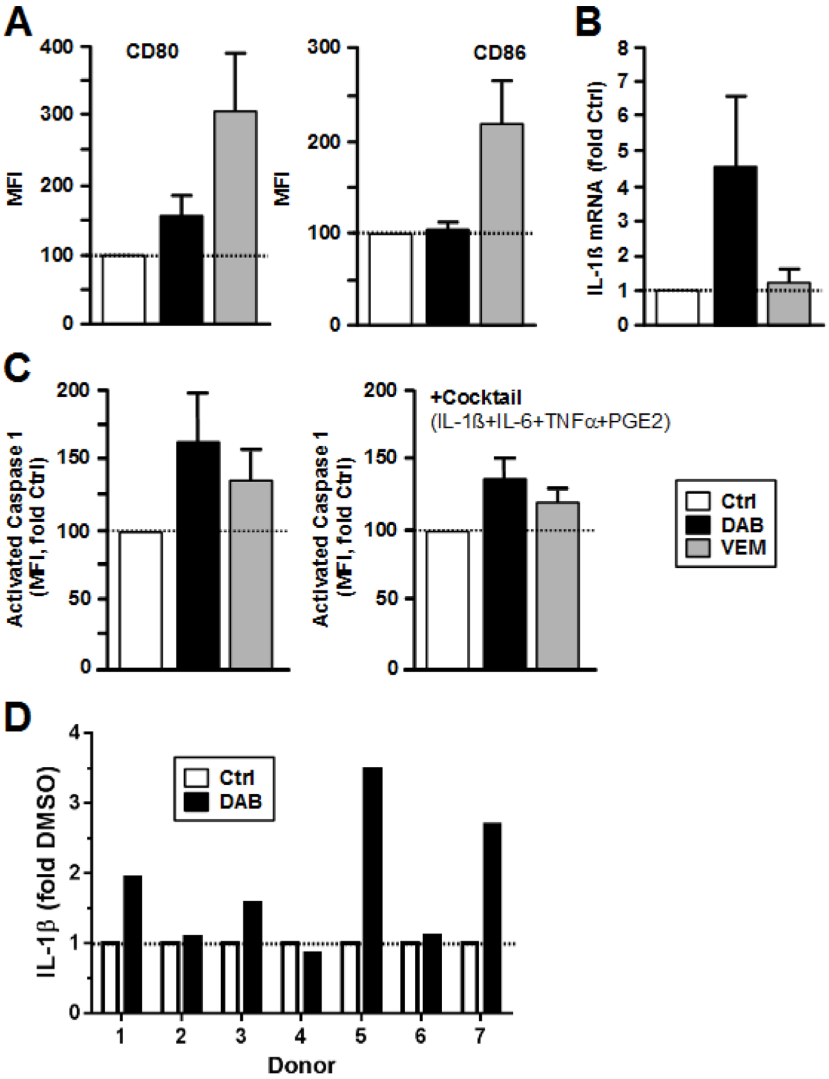

Supplementary Figure 5: VEM and DAB affect the phenotype of MODC, and DAB enhances IL-1ß production by human DC. (**A**–**C**) Unstimulated MODC on d6 of culture were treated with VEM and DAB (each 5 µM). DMSO (1‰) served as a solvent control (Ctrl). (A) After 48 h expression of CD80 and CD86 was assessed by flow cytometry. (B) IL-1ß mRNA levels were assessed by QPCR 6 h after onset of treatment, and were normalized to expression in the corresponding control. Data represent mean ± SEM of 3–4 independent experiments each. (C) Caspase 1 activity of MODC (*upper panel*) left unstimulated or (*lower panel*) cotreated with a maturation cocktail was measured by flow cytometry 6 h after application of $BRAF^{V600E}$ inhibitors. (A, C) Data show the MFI (mean ± SEM) of three independent experiments, normalized to the mean value of the Ctrl in each experiment. (**D**) Primary human DC immunomagnetically sorted from PBMC of different donors were treated overnight with DAB (2.5 µM) and DMSO (1‰) as control (Ctrl). IL-1ß amounts in DC supernatants were determined by CBA, and were normalized to IL-1ß contents of the corresponding Ctrl (basal IL-1ß expression in the different DC samples: 12.27 ± 5.60 ng/ml [mean ± SEM]).

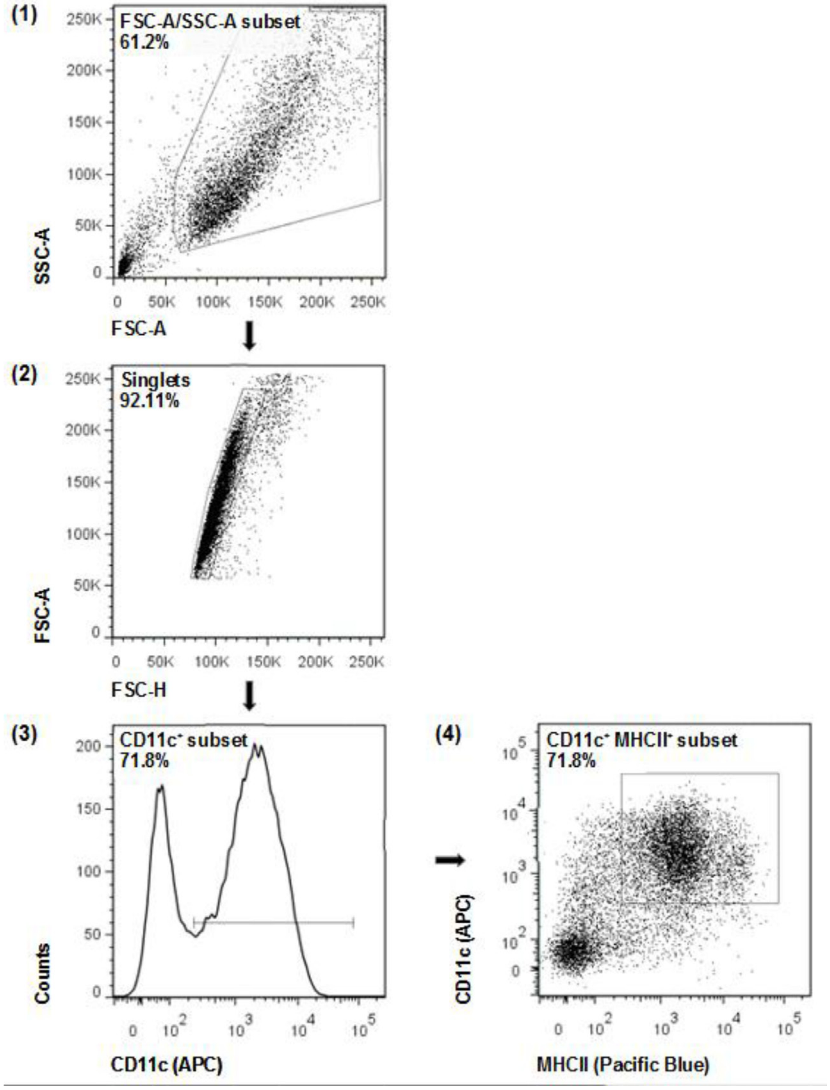

Supplementary Figure 6: Gating strategy for cytometric analysis of cell surface marker expression as exemplified for BMDC. (**1**) Exclusion of cell debris. (**2**) Exclusion of cell doublets. (**3**) Gating on $CD11c^+$ cells (DC lineage marker). (**4**) Further analysis on $CD11c^+$ cells for expression of MHCII via gating.
